# Peptide identification based on fuzzy classification and clustering

**DOI:** 10.1186/1477-5956-11-S1-S10

**Published:** 2013-11-07

**Authors:** Xijun Liang, Zhonghang Xia, Xinnan Niu, Andrew J Link, Liping Pang, Fang-Xiang Wu, Hongwei Zhang

**Affiliations:** 1 School of Mathematical Sciences, Dalian University of Technology, Dalian 116024, China; 2 Dept. of Computer Science, Western Kentucky University, Bowling Green, KY 42101, USA; 3 Dept. of Pathology, Microbiology and Immunology, Vanderbilt University School of Medicine, Nashville, TN 37232, USA; 4 Division of Biomedical Engineering, University of Saskatchewan, 57 Campus Dr., Saskatoon, SK S7N 5A9, Canada

**Keywords:** Peptide identification, Peptide spectrum matches (PSMs), Fuzzy support vector machine (SVM), Fuzzy silhouette

## Abstract

**Background:**

The sequence database searching has been the dominant method for peptide identification, in which a large number of peptide spectra generated from LC/MS/MS experiments are searched using a search engine against theoretical fragmentation spectra derived from a protein sequences database or a spectral library. Selecting trustworthy peptide spectrum matches (PSMs) remains a challenge.

**Results:**

A novel scoring method named FC-Ranker is developed to assign a nonnegative weight to each target PSM based on the possibility of its being correct. Particularly, the scores of PSMs are updated by using a fuzzy SVM classification model and a fuzzy silhouette index iteratively. Trustworthy PSMs will be assigned high scores when the algorithm stops.

**Conclusions:**

Our experimental studies show that FC-Ranker outperforms other post-database search algorithms over a variety of datasets, and it can be extended to solve a general classification problem with uncertain labels.

## Background

In protein identification, observed peptide spectra are searched against theoretical fragmentation spectra derived from target databases. Peptide spectrum matches (PSMs) are scored by database search tools and those high-scored PSMs are selected as target PSMs. In fact, more than half of selected PSMs are not correct [1]. Although many filters [2,3] have been proposed to refine the outputs further, they are not universal for different datasets.

To tackle this problem, PeptideProphet [4] used unsupervised learning for automatically selecting PSMs output by database search tools. Based on the assumption that the PSM samples are sampled from a mixture distribution which represents the chance of a "correct" PSM and an "incorrect" PSM, PeptideProphet applies the expectation maximization (EM) method to calculate the possibility of each PSM being "correct". As only the set of high-scored PSMs are searched for "correct" ones by PeptideProphet, some good low-ranked PSMs may be lost. Adaptive PeptideProphet was proposed in [5] to improve the performance of PeptideProphet by iteratively training a discriminant function from a set of top-ranked PSM samples, while [6] attempted to extend PeptideProphet by exploiting decoy PSMs in semi-supervised learning. In [7-9], decoy databases were used for validation of the performance of the post-database search algorithms. It is proposed in [6] to estimate a more accurate probability by combining decoy PSMs into a unified semi-supervised expectation- maximization framework.

Support vector machines (SVMs) have also been studied for the peptide assignment problem in [10,11]. Percolator [12] employed the SVM to iteratively adjust models fitting target PSMs with higher scores than decoy PSMs. Percolator, as a semi-supervised learning model, did not fully make use of the labels and samples of target PSMs. More recently, a fully supervised SVM learning model is proposed in [11] to improve the performance of Percolator by utilizing target PSM data, where those "incorrect" target PSMs are viewed as noises, and a special loss function is employed to reduce the noise's negative impact on the learning model. Although most good target PSMs are identified by the classification learning model from noises and decoy PSMs, all selected PSMs are treated in the same way.

In this paper, a new scoring method, FC-Ranker, is developed not only to identify reliable target PSMs, but also to evaluate the confidence of each target PSM. As good target PSMs are close to each other, FC-Ranker integrates sample clustering into the classification procedure to compute the possibility of each target PSM being correct. Compared with the standard SVM model, the proposed fuzzy classification model assigns a weight to each target PSM indicating its likelihood being correct. The score of each PSM sample is computed by combining discriminant function value and fuzzy silhouette value. The algorithm repeatedly updates the values of the discriminant function and fuzzy silhouette index for each PSM sample, and recompute the weights of targets until the algorithm stops. In experimental studies, while FC-Ranker shows a large overlap of the identified target PSMs with PeptideProphet and Percolator, it has identified more target PSMs in all datasets.

The first stage of the work was published in [13]. In this work, we compared the FC-Ranker algorithm with another benchmark method, Percolator, in the experimental studies. As Percolator is developed based on the SVM-based learning model, and hence it provides a better reference in performance comparison. Furthermore, we added a new dataset, Tal08, which has different characteristics (refer to Table 1) with datasets Yeast and UPS1. The performance of the proposed FC-Ranker algorithm has been conducted on all three datasets in terms of number of target PSMs, overlaps and ROC curves, and compared with PeptideProphet and Percolator. The new data analysis and results reinforce the efficiency of the proposed FC-Ranker method.

**Table 1:** Statistics of datasets

	Total	Target set				Decoy set			
			
		Total	Full	Half	None	Total	Full	Half	None
Yeast	14891	6702	1453	1210	4039	8189	106	1465	6618
UPS1	17335	8974	645	2013	6316	8361	118	1707	6536
Tal08	18653	9907	1081	2133	6693	8746	164	1923	6659

## Results and discussion

The FC-Ranker algorithm is compared with PeptideProphet [4] and Percolator [12] to validate its effectiveness. We used a PC with Intel (R) CPU 1.80 GHz×2, and RAM 2.0Gb for all experiments.

### Experimental Setup

#### Dataset

FC-ranker was examined over three datasets: S. cerevisiae Gcn4 (Yeast), Universal Proteomics Standard (UPS1) and Tal08 [14]. Trysin digestion of the protein samples generates three types of tryptic peptides: full-digested (both ends of a peptide satisfy enzyme specificity rule), half-digested (only one end satisfies the enzyme specificity rule) and none-digested (neither of the ends satisfies the rule). The database of Yeast protein sequences was obtained from Saccharomyes Genome Database (SGD) [15] and the Sigma48 protein sequences database from NCBI gene bank [16]. The attributes of each PSM sample include x-correlation, delta-cn, ions, sprank and calc-neutral-pep-mass.

The SEQUEST search results on UPS1 contains 48 purified human proteins and 17,335 PSMs, consisting of 8974 target PSMs and 8361 decoy PSMs. On the Yeast dataset, it contains 6652 proteins and 14,891 PSMs, consisting of 6702 target PSMs and 8189 decoy PSMs. On the Tal08 dataset, it contains 9907 target PSMs, and 8746 decoy PSMs, totally 18,653 PSMs.

Statistics of the three datasets are listed in Table 1.

#### Preprocess

In addition to those attributes output by SEQUEST, such as x-correlation, delta-cn, ions, sprank and calcneutral-pep-mass, another attribute "digested type" was added in the representation, with scalars "2", "1" and "0" for full-digested type, half-digested type, and none-digested type, respectively. The values of each attribute have been transformed linearly beforehand such that they have zero mean and unit variance (this is called a normalization process). We multiply a weight of 2.0 to the values of x-correlation and delta-cn attributes after normalization, inasmuch as these two attributes take more important position in data representation. As the attribute "digested type" also plays an important role by experimental experience, a weight of 2.0 was applied, similarly, on the values of this attribute after the normalization process.

#### Parameter setting

In all of the experiments, the parameter *c *is set to 1.0 in the proposed fuzzy linear programming SVM model where the Gaussian (RBF) kernel

$$k\left(x_{1},x_{2}\right)=\text{exp}\left(-\frac{||x_{1}-x_{2}|{|}^{2}}{2\sigma^{2}}\right),$$

was chosen, with parameter *σ *= 2.0.

In the iterations of FC-Ranker algorithm, we set *n *= 70 in Eq. (10) and $\hat{p}=0.03|{\text{Ω}}_{+}|$,$\hat{sep}=0.25$ Eq. (15). The strategy for solving large-scale programming was employed as described in the subsection "FC-Ranker for the large-scale problem", where the parameter *ρ *was chosen as 0.2.

### Validation of sep throughout iterations

**Figure 1:**
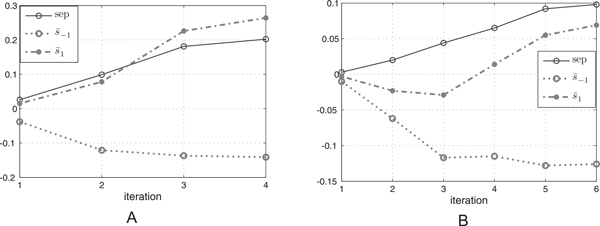
**Variations of *sep *throughout the iterations**. **A**: On Yeast dataset; **B**: On UPS1 dataset. The curve of *sep *is increasing throughout the iterations on both Yeast and UPS1 dataset. Similar curve of *sep *is also observed on Tal08 dataset, which is not listed here for simplicity of the layout.

Figure 1 depicts the variation of the values of *sep *in the iterations of the FC-Ranker algorithm on Yeast and UPS1 datasets. On both of the two datasets, the value of ${\bar{s}}_{1}$ is almost equal to ${\bar{s}}_{-1}$ initially, and then values of ${\bar{s}}_{1}$ increases as iterations proceed while values of ${\bar{s}}_{-1}$ decreases throughout the procedure. Hence, an increasing curve of *sep *which is defined as $\left({\bar{s}}_{1}-{\bar{s}}_{-1}\right)/2$is observed in the figure. At iteration 4 of Figure 1A(Yeast dataset) the value of *sep *exceeds the given threshold 0.25, reaching the termination criteria of the algorithm. The increasing values of *sep *illustrates that the identified good target PSMs indexed by Ω_1 _are closer to each other and were separated from decoy PSMs as the iterations increase, showing the effectiveness of the fuzzy silhouette index.

### Comparison of target PSMs

We compared the target PSMs output by PeptideProphet, Percolator and FC-Ranker under FDR level 0.05 in Table 2. On the Yeast, FC-Ranker identified 1475 target PSMs while PeptideProphet output 1443 target PSMs and Percolator output 1393 target PSMs. There are in all 32 target PSMs more found by FC-Ranker than PeptideProphet and 82 target PSMs more than Percolator. On the UPS1, there are 681 target PSMs found by FC-Ranker, which is 243 PSMs (55.5%) more than that of Percolator and 115 PSMs (20.3%) more than that of PeptideProphet. On the Tal08, FC-Ranker output 1092 target PSMs, which is 135 PSMs (14.1%) more than that of PeptideProphet and 139 PSMs (14.6%) more than that of Percolator. Similar results of PSMs output by the three methods on particular digested types are also shown in Table 2.

**Table 2:** Target PSMs output by PeptideProphet, Percolator and FC-Ranker

		TP+FP	TP				FP
				
			Total	Full	Half	None	Total
Yeast	PeptideProphet	1481	1443	1374	68	1	38
	Percolator	1429	1393	1342	51	1	36
	FC-Ranker	1513	1475	1376	83	16	38

UPS1	PeptideProphet	582	566	403	147	16	16
	Percolator	450	438	278	144	16	12
	FC-Ranker	698	681	444	198	39	17

Tal08	PeptideProphet	982	957	881	76	0	25
	Percolator	978	953	895	58	0	25
	FC-Ranker	1119	1092	865	173	54	27

**Figure 2:**
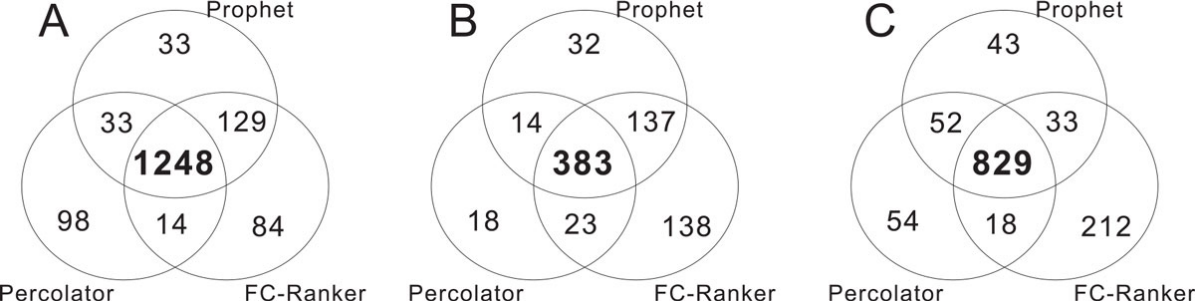
**Overlap of the identified PSMs by FC-Ranker, PeptideProphet and Percolator**. **A**: On Yeast dataset; **B**: On UPS1 dataset; **C**: On Tal08 dataset. "Prophet" indicates the results of PeptideProphet.

We analyzed the outputs of the target PSMs of the three methods and their overlaps are summarized in Figure 2. It is shown that there are large overlaps among the output PSMs of the three approaches in all Yeast, UPS1 and Tal08 datasets. Specifically, FC-Ranker, PeptideProphet and Percolator identified 1248 common target PSMs in Yeast dataset (Figure 2A), which covers 86.5% of the total target PSMs by PeptideProphet, 89.6% of the output of Percolator and 84.6% of the output targets of FC-Ranker. Particularly, FC-Ranker identified 129 PSMs (8.9%) selected by PeptideProphet but not covered by Percolator, and found 14 PSMs (1.0%) selected by Percolator but not covered by PeptideProphet.

On the UPS1 dataset (Figure 2B), the three algorithms have 383 target PSMs in common. The overlap covers 67.7% of the total target PSMs by PeptideProphet, 87.4% by Percolator and 56.2% by FC-Ranker. Particularly, there are 520 target PSMs catched by PeptideProphet and FC-Ranker in common, covering 91.9% of the total target PSMs by PeptideProphet and 76.4% by FC-Ranker; there are 406 target PSMs catched by Percolator and FC-Ranker in common, covering 92.7% of the total target PSMs by Percolator and 59.6% by FC-Ranker. Particularly, FC-Ranker identified 137 PSMs (24.2%) selected by PeptideProphet but not covered by Percolator, and found 23 PSMs (5.3%) selected by Percolator but not covered by PeptideProphet.

On the Tal08 dataset (Figure 2C), the three algorithms have 829 PSMs in common. The overlap covers 86.6% of the total target PSMs by PeptideProphet, 87.0% by Percolator and 75.9% by FC-Ranker. Particularly, there are 862 target PSMs catched by PeptideProphet and FC-Ranker in common, covering 90.1% of the total target PSMs by PeptideProphet and 78.9% by FC-Ranker; there are 847 target PSMs catched by Percolator and FC-Ranker in common, covering 88.9% of the total target PSMs by Percolator and 77.6% by FC-Ranker. Particularly, FC-Ranker identified 33 PSMs (3.4%) selected by PeptideProphet but not covered by Percolator, and found 18 PSMs (1.9%) selected by Percolator but not covered by PeptideProphet.

### ROC curve

**Figure 3:**
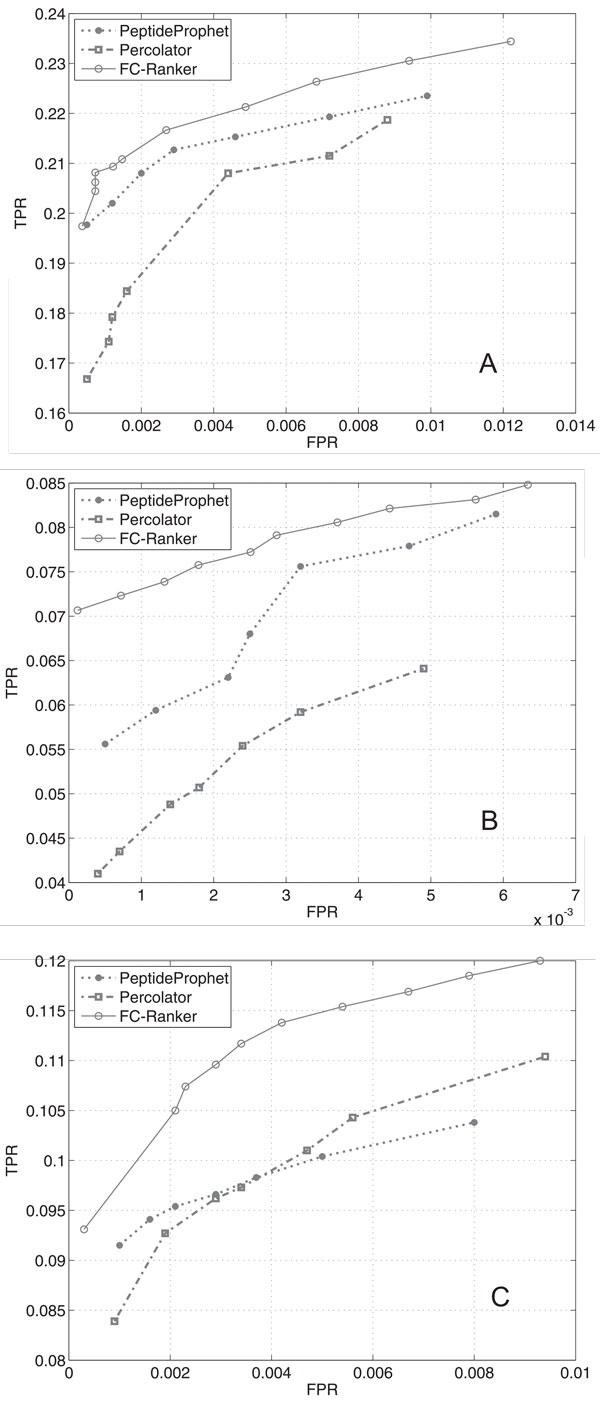
**ROC curves of FC-Ranker, PeptideProphet and Percolator**. **A**: On Yeast dataset; **B**: On UPS1 dataset; **C**: On Tal08 dataset. True Positive Rate (TPR): *TPR = TP/*(*TP *+ *FN*), False Positive Rate (FPR): *FPR = FP/*(*FP *+ *TN*), with *TP *: number of true positives, *FP *: number of false positives, *FN *: number of false negatives, *TN *: number of true negatives.

Figure 3 shows ROC curves of the three methods on the Yeast, UPS1 and Tal08 datasets. On the Yeast dataset (Figure 3A), when FPR level near zero FC-Ranker has the same TPR level with PeptideProphet while higher TPRs are reached by FC-Ranker than those by PeptideProphet and Percolator on other FPR levels. On both the UPS1 dataset (Figure 3B) and Tal08 dataset (Figure 3C), FC-Ranker reaches higher TPRs than the other two methods throughout all FPR levels. Particularly, on Tal08 dataset, FC-Ranker reaches evidently high TPR levels even on comparatively high FPR levels.

**Figure 4:**
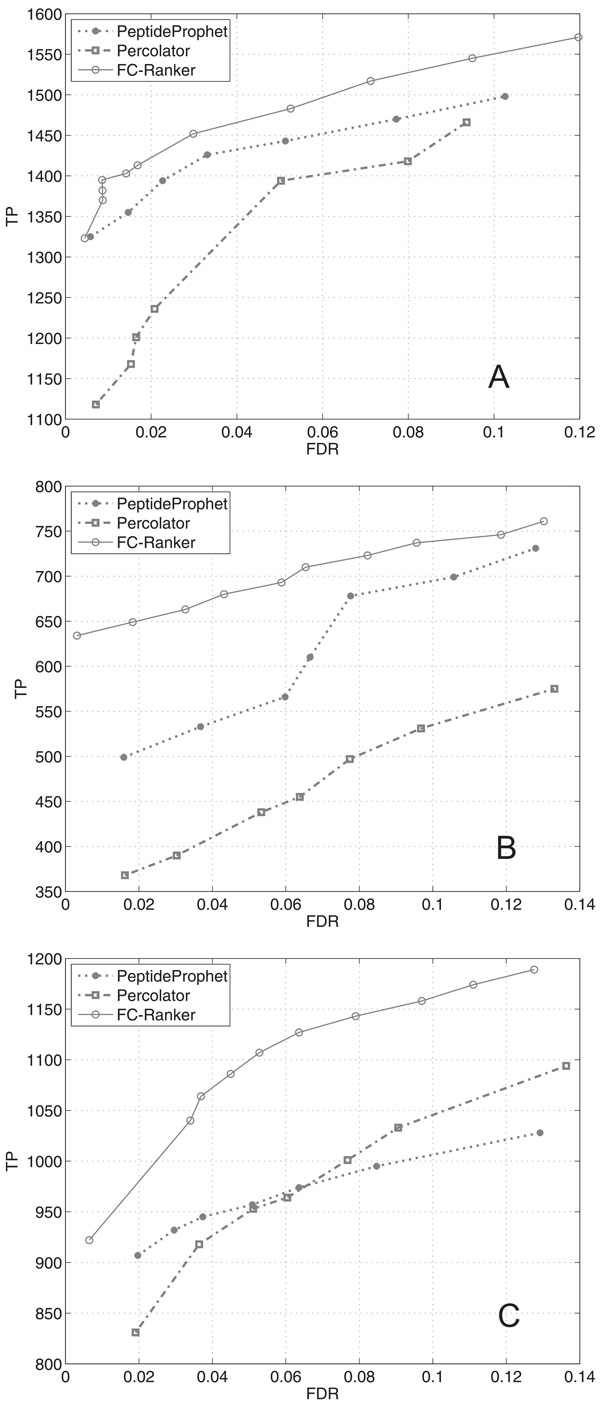
**Performance comparison of FC-Ranker, PeptideProphet and Percolator in terms of the number of true positives (TPs)**. **A**: On Yeast dataset; **B**: On UPS1 dataset; **C**: On Tal08 dataset. False Discovery Rate (FDR): *FDR *= 2 *· FP/*(*FP *+ *TP*), with *TP *: number of true positives, *FP *: number of false positives.

Figure 4 depicts the relation between the number of TP and FDR, where we observed similar patterns with the corresponding ROC curves.

## Methods

### Classification and clustering methods for peptide identification

#### Fuzzy clustering

Clustering analysis is an unsupervised learning method to group similar data samples together. Silhouette index was introduced in [17,18] to measure how well a sample belongs to a cluster.

Suppose that there are *l *data samples {*x*_1_, . . ., *x_l_*}, which are grouped into *K *clusters, denoted as *C *={*C*_1_*, . . ., C_K_*}. Denote by *d*(*x_i_, x_j_*) the distance between two samples *x_i _*and *x_j_*, and by $C_{k}=\left\{x_{1}^{k},\ldots ,x_{mk}^{k}\right\}$ the samples of the *k*th cluster, where *m_k _= |C_k _| *and *k *= 1*, . . ., K*. The average distance, denoted by $a_{i}^{k}$, between the *i*th data sample in cluster *C_k_*and other samples in the same cluster is formulated as

$$a_{i}^{k}=\frac{1}{m_{k}-1}\underset{j=1,\ldots ,m_{k},j\neq i}{\sum }d\left(x_{i}^{k},x_{j}^{k}\right),\;i=1,\ldots ,m_{k},$$

and the minimum average distance between the *i*th data sample in cluster *C_k_*and all other data samples in clusters *C_v_, v *= 1*, . . ., K, v *≠ *k *is defined as

$$b_{i}^{k}=\underset{v=1,\ldots ,K,v\neq k}{\text{min}}\left\{\frac{1}{m_{v}}\overset{m_{v}}{\underset{j=1}{\sum }}d\left(x_{i}^{k},x_{j}^{v}\right)\right\},\;i=1,\ldots m_{k}.$$

Then, we define the silhouette value of the *i*th data sample in *C_k _*as follows

$$s_{i}^{k}=\frac{b_{i}^{k}-a_{i}^{k}}{\text{max}\left\{a_{i}^{k},b_{i}^{k}\right\}}.$$

Clearly, the silhouette values located in the interval [*−*1, 1]. The silhouette value of the cluster *C_k _*is defined as

$$s_{k}=\frac{1}{m_{k}}\overset{m_{k}}{\underset{i=1}{\sum }}s_{i}^{k},\;k=1,\ldots ,K.$$

#### Classification

Our task is to identify those correct PSMs from a set of PSMs generated by some database searching tools in peptide identification. Usually decoy PSMs are employed to validate target PSMs, then the samples of PSMs can be categorized into "good" class, with labels " +1", and "bad" class, with labels "*−*1". In the setting of classification, we use a vector of attributes such as x-correlation, delta-cn, ions, sprank, calc-neutral-pepmass, etc., to represent a PSM data sample. Let {*x_i_*} ⊆ *R^q^, i *= 1*, . . ., l *be the PSM data samples with *q *the number of attributes. We aim at finding a discriminant function *f *: *R^q ^→ R *to classify the PSM data samples according to their labels.

One of the greatest challenges arising from the problem of the peptide identification is that there is lack of data samples with deterministic +1 labels. For a standard classification setting, the discriminant function is solved by training the models on two balanced types of data samples with deterministic labels. In peptide identification problem, however, a great number of PSMs generated by database searching engines are incorrect, and the data samples with +1 labels are quite unreliable. Thus, the great amount of data samples with incorrect +1 labels would extremely distort the trained discriminant function if they are employed directly in the standard classification models.

Here, we consider the kernel-based SVM classifier as follows:

$$f\left(x\right)=\overset{l}{\underset{i=1}{\sum }}\alpha_{j}k\left(x_{j},x\right)+b$$

where *b *∈ *R, k*(*·,·*) is a chosen kernel function. The label of a data sample *x *is predicted as +1, if *f *(*x*) *>*0, otherwise it is predicted as −1. A quadratic programming is usually solved to obtain the coefficients *α *and *b*, which requires huge computations overhead, especially for large-scale problems. To overcome this problem, a class of linear programming SVM is introduced in [19].

For the *l *data samples {(*x_i_, y_i_*)}, *i *= 1*, . . ., l*, with *x_i _*∈ *R^q^, y_i _*∈ {1, −1}, the linear programming SVM model is formulated as

(1) $$\begin{array}{cc} \underset{\alpha ,r,\xi ,b}{\text{min}} & -r+c\sum_{i=1}^{l}\xi_{i}\\ \text{s}\text{.t}\text{.} & yif\left(x_{i}\right)=y_{i}\left(\sum_{j=1}^{l}\alpha_{j}y_{j}k\left(x_{j},x_{i}\right)+b\right)\geq r-\xi_{i},\\ & -1\leq \alpha_{i}\leq 1,\xi_{i}\geq 0,\;i=1,\ldots ,l \end{array}$$

where *c >*0 is a given constant, and the discriminant function $f\left(\cdot \right)=\sum_{j=1}^{l}\alpha_{j}y_{j}k\left(x_{j},\cdot \right)+b$.

### The basic FC-Ranker algorithm

In this section, the FC-Ranker algorithm is present to calculate the score of each PSM data sample. The score values reflect the possibility of the PSM data samples being correct, and those PSMs with high scores are selected for users at last.

**Figure 5:**
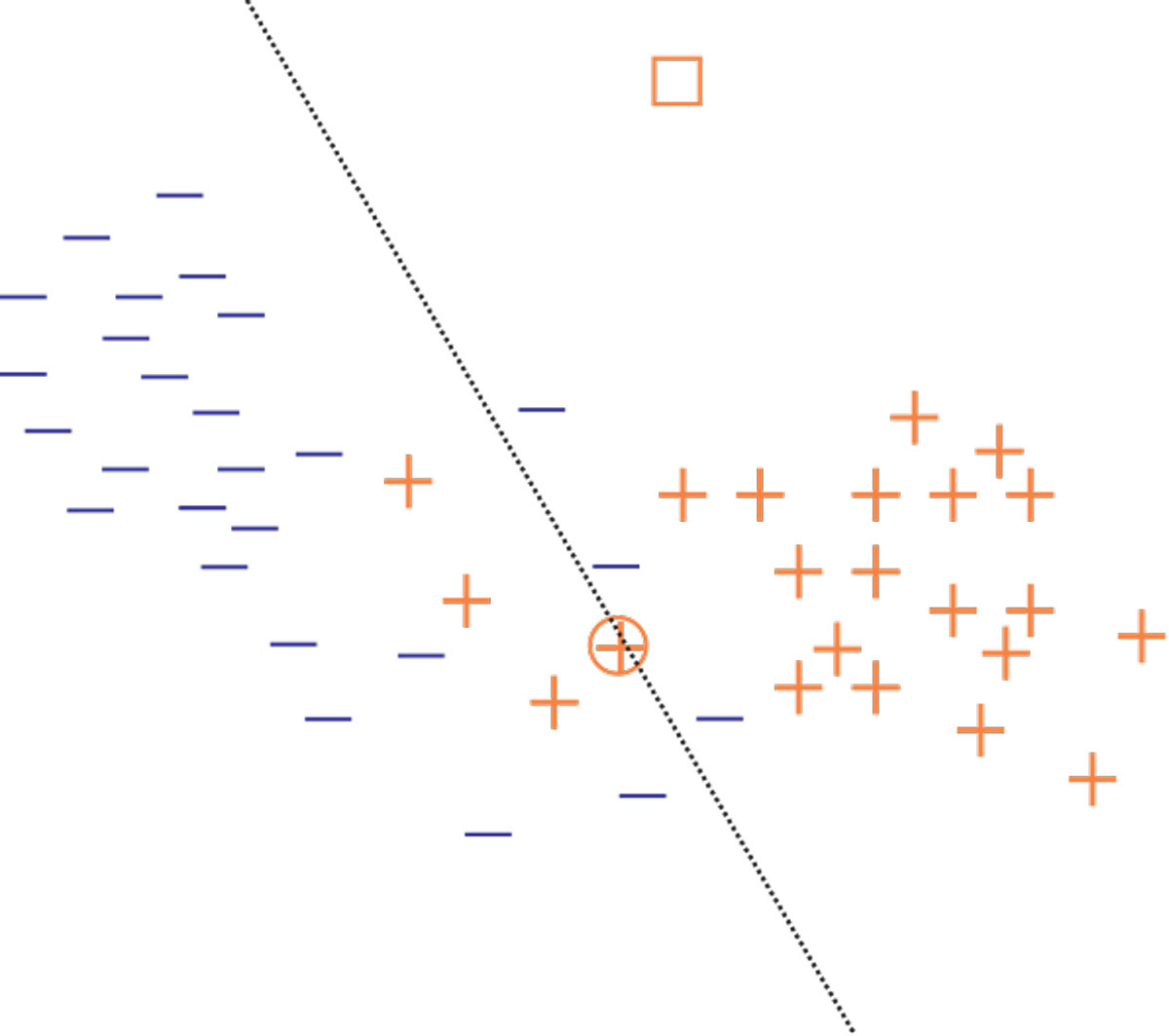
**Classification and clustering**. "−" represents decoy PSM, while "+" represents target PSM. The data sample represented by "□" locates far away from the decision boundary. However, the possibility of it being a correct PSM is remote since it goes too far away from other data target PSMs. The data sample represented by "⊕" also has a small possibility to be a correct PSM since it locates near the decision boundary.

Denote by Ω = {1*, . . ., l*} the set of indices of *l *PSM data samples, by Ω_+ _the set of indices of target PSMs, by

$${\text{Ω}}_{-1}=\left\{i\in \text{Ω}|y_{i}=-1\right\},$$

the set of indices of decoy PSMs, by Ω_1 _the set of indices of good target PSMs, and Ω_0 _= Ω_+ _*\ *Ω_1 _the set of bad target PSMs. The FC-Ranker algorithm aims to select the set Ω_1 _from Ω_+ _utilizing the data samples indexed by Ω*_−_*. To classify good target PSMs from others, a discriminant function *f *is constructed such that the function value *f *(*x_i_*) is positive if sample *x_i _*belongs to Ω_1_, and negative otherwise. A large discriminant function value of a target PSM sample *x_i _*indicates that the sample locates far away from the decision boundary, and hence large possibility of being a good PSM. However, only a large discriminant function value of *f *(*x_i_*) itself is not sufficient to ensure that the PSM sample *x_i _*is good. Take the sample represented by "□" in Figure 5 as an example, it has a large distance from the decision boundary and thus has a large function value of *f *(□). This sample, however, tends to be a bad PSM since it locates too far away from the other PSM data samples indicated by the set Ω_+_.

On the other hand, a data sample may not be a good target PSM either if it locates comparatively close to other target PSMs but has a small discriminant function value. The data sample represented by "⊕" in Figure 5 should also be excluded from the set Ω_1_. The above observations hints us that a good target PSM data sample should satisfy two rules: 1) has a large discriminant function value; 2) is close to other target PSMs.

#### Fuzzy SVM classification

A weight *θ_i _*∈ [0, 1] is introduced for each target sample *x_i _*indexed by Ω_+ _to indicate its possibility of being correct since its label is not trustworthy. A large weight of a sample usually indicates that the PSM has more possibility to be correct. Since it is definitely sure that the decoy PSMs are incorrect, we constantly set the weights *θ_i _*to 1 for *x_i _*∈ Ω*_−_*. Denote *loss*(*f *(*x_i_*), *y_i_*) the empirical error of sample *x_i_*, then the empirical error can be formulated as $\underset{i\in \text{Ω}}{\sum }\text{ loss}(f(x_{i}),y_{i})$ in traditional classification problems with deterministic labels. Assigning a weight to each data sample, we reformulate the total empirical error as $\underset{i\in \text{Ω}}{\sum }\theta_{i}\text{ · loss}(f(x_{i}),y_{i})$.

Thus, the linear programming SVM model (1) is transformed as follows

(2) $$\begin{array}{cc} \underset{\alpha ,r,\xi ,b}{\text{min}} & -r+c\sum_{i\in \text{Ω}}\theta_{i}\xi_{i}\\ \text{s}\text{.t}\text{.} & y_{i}\left(\sum_{j=1}^{l}\alpha_{j}y_{j}k\left(x_{j},x_{i}\right)+b\right)\geq r-\xi_{i},i\in \text{Ω},\\ & -1\leq \alpha_{i}\leq 1,\xi_{i}\geq 0,\;i\in \text{Ω}, \end{array}$$

where *α *∈ *R^l^, b *∈ *R*^1^, *r *∈ *R*^1 ^and *ξ *= [*ξ*_1_*, . . ., ξ_l_*] ∈ *R^l^*. Model (2) is referred as the fuzzy linear programming SVM model.

The model (2) can be rewritten as

(3) $$\begin{array}{cc} \underset{\alpha ,r,\xi ,b}{\text{min}} & \left\langle \left[0_{t}^{T}\;0\;\;c\theta^{T}-1\right],\;\;\left[\alpha^{T}b\;\xi^{T}r\right]\right\rangle \\ \text{s}\text{.t}\text{.} & \left[\text{Λ}\left(y\right)K\text{Λ}\left(y\right)\;y\;I_{l}-1_{l}\right]\left[\begin{array}{c} \alpha \\ b\\ \xi \\ r \end{array}\right]\geq 0,\\ & r\geq 0,\\ & -1\leq \alpha_{i}\leq 1,\xi_{i}\geq 0,\;i\in \text{Ω}, \end{array}$$

where *θ *= [*θ*_1_, . . ., *θ_l_*]*^T ^*, Λ(*y*) = *Diag*(*y*), 0*_l _*∈ *R^l ^*is a vector with zero elements, 1*_l _*∈ *R^l ^*is a vector with each element equal to 1, *I_l _*is the *l × l *unit matrix, and *K *= (*k*(*x_i_, x_j_*))_1*≤i≤l*,1*≤j≤l*_. The model can be solved by existing optimization softwares, such as Mosek.

#### Fuzzy silhouette

To adapt the situations with uncertain labels we generalize the silhouette concept for deterministic setting to fuzzy silhouette index.

For *k = −*1, 1, *i *∈ Ω*_k_*, the average distance of sample *x_i _*to the other data samples in Ω*_k _*is formulated as

(4) $$\beta_{i}^{k}=\frac{\sum_{j\in {\text{Ω}}_{k},j\neq i}^{}\theta_{j}d\left(x_{i},x_{j}\right)}{\sum_{j\in {\text{Ω}}_{k},j\neq i}^{}\theta_{j}}$$

where *θ_i _*∈ [0, 1]. Then, we define the *fuzzy silhouette *of sample *x_i _*as

(5) $$s_{i}=\frac{\beta_{i}^{-1}-\beta_{i}^{1}}{\text{max}\left\{\beta_{i}^{-1},\beta_{i}^{1}\right\}},\;i\in \text{Ω}.$$

It measures the degree that a PSM sample goes far away from the decoys and that is close to the good target samples. Hence, a PSM data sample is more likely to be a correct one if it has a large fuzzy silhouette value.

For the sets of Ω_-1_, Ω_1 _and Ω_0 _we define their average fuzzy silhouettes as

$${\bar{s}}_{k}=\frac{\sum_{i\in {\text{Ω}}_{k}}^{}s_{i}}{\left|{\text{Ω}}_{k}\right|}$$

where *|*Ω*_k _| *is the cardinality of Ω*_k _, k = −*1, 1, 0. We also define

(6) $$sep=\left({\bar{s}}_{1}-{\bar{s}}_{-1}\right)/2$$

as a metric to indicate the separation degree of decoy PSM samples and good PSMs.

#### Score of the samples

Based on the fuzzy SVM model and fuzzy silhouette metric we design a scoring scheme, which defines the score of sample *x_i _*as

(7) $$score\left(i\right)=\left(1-sep\right)\cdot \varphi \left(f\left(x_{i}\right)\right)+sep\cdot \psi \left(s_{i}\right),$$

where *φ*(*·*) and *ψ*(*·*) are functions for scaling the values of *f *(*x_i_*) and *s_i_*, respectively. Here, function *φ*(*·*) : *R → *[*−*1, 1] is constructed as an increasing function, and *ψ*(*·*) as an increasing function mapping from [*−*1, 1] to [*−*1, 1]. Particularly, we choose function *φ*(*f *(*x_i_*)) and *ψ*(*s_i_*) as

(8) $$\varphi \left(f\left(x_{i}\right)\right)=\frac{2}{\pi }sign\left(f\left(x_{i}\right)-f_{0}\right)\text{atan}\left({\left(|f\left(x_{i}\right)-f_{0}|f_{\text{max}}\right)}^{1/4}\right),$$

(9) $$\psi \left(s_{i}\right)=\left(s_{i}-s_{0}\right)/s_{\text{max}},$$

where *f*_max _and *s*_max _are the largest values of {*|f *(*x_i_*) *− f*_0_*|*} and {*|s_i _− s*_0_*|*} for *i *∈ Ω_+_, respectively, and *f*_0 _is the threshold of the values of discriminant function, *s*_0 _the threshold of fuzzy silhouette. The power of $\frac{1}{4}$ on *|f *(*x_i_*) *− f*_0_*| *is introduced to smooth the weight contributions.

#### The FC-Ranker algorithm

The FC-Ranker algorithm iteratively adjusts the index set of good PSM Ω_1 _by calculating the scores and weights of the data samples until a stop criterion is met. Initially, the algorithm set ${\text{Ω}}_{1}^{0}={\text{Ω}}_{+}$ and ${\text{Ω}}_{0}^{0}=\phi$, i.e. all PSM samples are viewed as good ones at iteration 0. At iteration *k*, the algorithm solves the fuzzy linear programming SVM model (3), calculates the fuzzy silhouette values of the samples according to Eq. (5) and updates the index set Ω_1 _and Ω_0 _such that the indices of target PSMs in Ω_1 _with small scores are moved to Ω_0_, while the indices of target PSMs in Ω_0 _with large scores are moved to Ω_1_.

At the *k*th iteration, PSM samples indexed by Ω_+ _are ranked according to their scores, and the top *n*% of them in Ω_1 _are reserved. Then ${\text{Ω}}_{1}^{k}$ is updated by the discriminant function values as

(10) $${\text{Ω}}_{1}^{k+1/3}=\left\{i\in {\text{Ω}}_{1}^{k}|f\left(x_{i}\right)\;\text{is ranked at top}\;n\%\text{ in all}\;{\left\{f\left(x\right)\right\}}_{i\in {\text{Ω}}_{1}^{k}}\right\},$$

where 0 *< n <*100 is a constant percentage. Based on the calculated fuzzy silhouettes, ${\text{Ω}}_{1}^{k+1/3}$ is then updated by

(11) $${\text{Ω}}_{1}^{k+2/3}=\{i\in {\text{Ω}}_{1}^{k+1/3}|s_{i}\text{ is ranked at top }n\%\text{ in all}\;{\left\{s_{j}\right\}}_{j\in {\text{Ω}}_{1}^{k+1/3}}\}$$

and ${\text{Ω}}_{0}^{k}$ is updated by

(12) $${\text{Ω}}_{0}^{k+1/3}={\text{Ω}}_{+}\backslash {\text{Ω}}_{1}^{k+2/3}.$$

Finally, for *i *∈ Ω new scores *score*(*i*)^*k*+1^, are computed according to Eq. (7) and the weights $\theta_{i}^{k+1}$ are calculated by the following equation

(13) $$\theta_{i}^{k+1}\text{ = }\left\{\begin{array}{cc} \text{max}\left\{score{\left(i\right)}^{k+\text{1}},0\right\}, & i\in {\text{Ω}}_{+};\\ 1, & i\in {\text{Ω}}_{-.} \end{array}\right)\;$$

Then indices of the samples indexed by ${\text{Ω}}_{0}^{k+1/2}$ are moved to ${\text{Ω}}_{1}^{k+2/3}$ if the samples have large score values,

i.e.,

(14) $$\begin{array}{c} {\text{Ω}}_{1}^{k+1}={\text{Ω}}_{1}^{k+2/3}\cup \left\{i\in {\text{Ω}}_{0}^{k+1/2}|f\left(x_{i}\right)\geq {\bar{f}}_{1}^{k+2/3}\right\},\\ {\text{Ω}}_{0}^{k+1}={\text{Ω}}_{+}\backslash {\text{Ω}}_{1}^{k+1}, \end{array}$$

where ${\bar{f}}_{1}^{k+2/3}$is the average of $\left\{f\left(x_{i}\right)|i\in {\text{Ω}}_{1}^{k+2/3}\right\}$.

The algorithm terminates when the number of identified good PSM samples reaches a given threshold $\overset{⌢}{p}$, or the separation degree *sep*^*k*+1 ^defined by Eq. (6) reaches a threshold $\hat{sep}$, i.e.,

(15) $$\left|{\text{Ω}}_{1}^{k+1}\right|\leq \overset{⌢}{p},\;\text{or}\;sep^{k+1}\geq \hat{sep.}$$

The FC-Ranker algorithm is summarized in Algorithm 1.

**Algorithm 1 **The FC-Ranker Algorithm

**Input: **{*x_i_, y_i_*}, *i *∈ Ω;

**Output: **Scores of samples indexed by Ω;

1: Initialization: *k = −*1, ${\text{Ω}}_{1}^{0}={\text{Ω}}_{+}$, ${\text{Ω}}_{0}^{0}: =\text{Ø}$, $\theta_{i}^{0}=1$, *i *∈ Ω.

2: **while **Stop criterion (15) is not satisfied **do**

3:    *k *:= *k *+ 1.

4:    SVM classification.

5:        Solve fuzzy SVM classification model Eq. (3);

6:        Calculate ${\text{Ω}}_{1}^{k+1/3}$ via Eq. (10).

7:    Clustering analysis.

8:        Calculate fuzzy silhouettes *s_i_, i ∈ *Ω via (5);

9:        Calculate ${\text{Ω}}_{1}^{k+2/3}$, ${\text{Ω}}_{0}^{k+1/2}$ via Eq. (11), (12).

10:    Update weights.

11:        Calculate *score*(*i*)^*k*+1^, *θ*^*k*+1 ^via Eq. (7), (13);

12:        Calculate ${\text{Ω}}_{1}^{k+1}$, ${\text{Ω}}_{0}^{k+1}$, *sep*^*k*+1 ^via Eq. (14), (6).

13: **end while**

### FC-Ranker for the large-scale problem

The number of PSMs output by a database search engine is usually extremely large. In this section, some implementation practice is discussed further such that the algorithm is capable for solving large-scale problems.

#### Fuzzy SVM classification for the large-scale problem

If the data matrix is sparse, the interior-points algorithms would be competent in solving large-scale linear programming problems. The kernel matrix *K *in Problem (3) is, unfortunately, not sparse in general. In fact, kernel matrix *K *is usually quite dense and most of its elements are nonzero. To store a large dense matrix *K *is not a trivial task. Take a matrix *K *with Gaussian kernel and *l *= 400, 000 as an example, if four bytes are occupied per element then the matrix *K *would have *l*^2 ^= 1.6 *× *10^11 ^elements and take up 640Gb of storage in all.

Interestingly, our experimental experience indicates that the kernel matrix is usually quite low rank in the peptide identification problem. Hence, a sub-matrix *K' *consisting of *l' *columns of *K *(*l' << l*) is selected to substitute *K *in Problem (3). These *l' *columns of the sub-matrix are selected randomly from the total columns of matrix *K*. This operation can be implemented by sampling *l' *data samples randomly and then calculating the sub-matrix *K' *according to the kernel function. It reduces the storage greatly. Denote an index set Ω*' ⊂ *Ω which consists of the indices of *l' *columns. Then the matrix (*K'*)*_ij _= k*(*x_i_, x_j _*)*, i ∈ *Ω*, j ∈ *Ω*' *can be calculated with size of *l × l'*. Let *y' *= (*y'*)_*j∈*Ω_', then Problem (3) is reduced to

(16) $$\begin{array}{cc} \underset{\alpha ,r,\xi ,b}{\text{min}} & \left\langle \left[0_{t}^{T}\;0\;c\theta^{T}-1\right],\left[\alpha^{T}b\;\xi^{T}r\right]\right\rangle \\ \text{s}\text{.t}\text{.} & [\text{Λ}\left(y\right)K^{\prime }\text{Λ}\left(y^{\prime }\right)\;y\;I_{l}-1_{l}]\;\left[\begin{array}{c} \alpha \\ b\\ \xi \\ r \end{array}\right]\geq 0,\\ & r\geq 0,\xi_{i}\geq 0,\;i\in \text{Ω}\\ & -1\leq \alpha_{i}\leq 1,\;j\in {\text{Ω}}^{\prime }. \end{array}$$

Where $\alpha \in R^{l^{\prime }}$, *b *∈ *R*^1^, *r *∈ *R*^1^, *r *∈ *R^l^*, and Λ(*y′*) = *Diag*(*y′*).

### Fuzzy silhouette for the large-scale problem

For updating fuzzy silhouette value *s_i _*of sample *i*, the major work is to compute $\beta_{i}^{1}$ and $\beta_{i}^{-1}$ in Eq. (4) where it is required to calculate *l *distances. In all, each iteration computes *|*Ω| * *|*Ω*| = l*^2 ^distances with total samples. Denote a given sample rate by *ρ *with *ρ *∈ (0, 1). We sample ρ * *|*Ω_1_*| *indices of targets from Ω_1_, and ρ * *|*Ω_*−*1_*| *indices of decoys from Ω_*−*1_, denoted by Ω*t *and ${\text{Ω}}_{-1}^{\prime }$, to substitute Ω_1 _and Ω_−1 _in Eq. (4), resp. Then at most *ρl*(*|*Ω_*−*1_*| *+ *|*Ω_1_*|*) *≤ ρl *distances need to be calculated at each iteration.

## Conclusion

A new scoring method has been developed based on the iterations of FC-Ranker algorithm which were equipped with fuzzy silhouette index and a fuzzy SVM classification model to cope with the large amount of incorrect labels of target PSM samples. In the fuzzy classification model, each PSM was assigned a calculated weight which indicates the possibility of the PSM sample being correct. The performance of FC-Ranker algorithm has been compared with PeptideProphet and Percolator on Yeast, UPS1 and Tal08 datasets, showing that FC-Ranker surpassed PeptideProphet and Percolator in terms of ROC and the quantity of identified target PSM samples under the same FDR level. Moreover, FC-Ranker outputs more target PSMs than PeptideProphet and Percolator does while they share a large number of PSMs in common.

## Abbreviations

PSMs: peptide spectrum matches; SVM: support vector machine

## Competing interests

The authors declare that they have no competing interests.

## Authors' contributions

XL and ZX designed the basic FC-Ranker algorithm and wrote the manuscript. XN, AL and FW designed the version of FC-Ranker algorithm for the large-scale problem and corresponding experiments. XL, LP and HZ designed and operated experiments. All authors read and approved the final manuscript.

## Declarations

The publication costs for this article were funded by Xijun Liang.

This article has been published as part of *Proteome Science *Volume 11 Supplement 1, 2013: Selected articles from the IEEE International Conference on Bioinformatics and Biomedicine 2012: Proteome Science. The full contents of the supplement are available online at http://www.proteomesci.com/supplements/11/S1.
